# The CURB65 score predicted 180-day mortality of non-small cell lung carcinoma patients with immune checkpoint inhibitor-associated pneumonitis: A pilot retrospective analysis

**DOI:** 10.3389/fonc.2022.927858

**Published:** 2022-08-01

**Authors:** Fen Lan, Bo Fan, Lihua Wang, Lixia Xia, Ting Zhang, Wen Li, Yanxiong Mao

**Affiliations:** ^1^ Key Laboratory of Respiratory Disease of Zhejiang Province, Department of Respiratory and Critical Care Medicine, Second Affiliated Hospital of Zhejiang University School of Medicine, Hangzhou, China; ^2^ Department of Respiratory and Critical Care Medicine, First People’s Hospital of Jiashan, Jiashan, China; ^3^ Department of Radiology, Second Affiliated Hospital of Zhejiang University School of Medicine, Hangzhou, China; ^4^ Department of Radiotherapy, Second Affiliated Hospital of Zhejiang University School of Medicine, Hangzhou, China

**Keywords:** checkpoint inhibitor-associated pneumonitis, non-small cell lung carcinoma, CURB65, mortality, adverse events - complications

## Abstract

**Introduction:**

The immune checkpoint inhibitor-associated pneumonitis (CIP) is a particularly worrisome and potentially lethal form of immune-related adverse events. An objective and evidence-based assessment tool for evaluating the severity of CIP is in urgent need. CURB65 (consciousness, urea nitrogen, respiratory rate, blood pressure, and age) is a potential candidate to meet the need.

**Methods:**

A retrospective study was conducted to explore preliminarily if CURB65 could predict the mortality in non-small cell lung carcinoma (NSCLC) patients with CIP.

**Results:**

A total number of 28 NSCLC patients with CIP were included in the current study and classified into low-CURB65 group (n = 21) and high-CURB65 group (n = 7). Mortality after onset of CIP was consistently higher in the high-CURB65 group than in the low-CURB65 group (30-day: 57.1% vs. 0; 90-day: 71.4% vs. 4.76%; 180-day:71.4% vs. 14.29%). Two patients (9.5%) in the low-CURB65 group had severe CIP, and more than half of patients in the high-CURB65 group had severe CIP (p = 0.0008). The patients in the high-CURB65 group received more aggressive treatment. Both groups showed a predominant organizing pneumonia-like pattern on CT scan. CURB65 was moderately correlated with the American Society of Clinical Oncology (ASCO) grade of CIP, with a Pearson correlation coefficient R of 0.524.

**Conclusion:**

CURB65 accurately stratified the risk of mortality in NSCLC patients with CIP. CURB65 might complement the ASCO grade in the assessment and prediction of mortality in these populations.

## Introduction

Immune checkpoint inhibitors (ICIs) have revolutionized non-small cell lung carcinoma (NSCLC) treatment and became a first-line treatment in advanced and locally advanced NSCLC without driver gene alterations (1–3). With expanded use of ICIs, the unique immune-related adverse events (irAEs) have been increasingly reported (4, 5). As the particularly worrisome and potentially lethal form of irAEs, checkpoint inhibitor-associated pneumonitis (CIP) has drawn increasing attention (6–9). CIP is characterized by the occurrence of respiratory symptoms/signs related to a new emerging infiltration viewed on a chest imaging but excluding new infections or alternative etiologies (10). The reported incidence of CIP in NSCLC ranges from 2% to 38% in clinical trials, and from 4.8% to 39.3% in real-world studies (11).

At present, there is no consensus on the diagnostic evaluation, risk stratification, and optimal management of CIP, which are significant barriers to improved prognosis (12). Because the clinical appearance of CIP varies widely from mild symptoms to severe dyspnea and respiratory failure, it is generally accepted that treatment should be personalized and depend on the severity of CIP (6, 11). Currently, the severity of CIP is usually graded according to clinical symptoms and/or imaging manifestations (13–15). Because clinical symptoms are essential for CIP grade, a considerable level of subjectivity is inevitable. It is possible that patients and their clinicians have different perceptions of the bother caused by different symptoms. Clinicians may have disagreement in the grade for the same CIP and treat the patient differently, which might influence the final outcome. Moreover, all these recommendations are expert consensus based, with benefits outweighing harms, and strengths of recommendations are only moderate. Therefore, an objective and evidence-based assessment tool for evaluating the severity of CIP is in urgent need.

Community-acquired pneumonia (CAP) refers to the infectious inflammation of lung parenchyma acquired outside of hospitals (16, 17). CIP and CAP, while differing in etiologies, present with similar symptoms such as fever, cough, sputum production, chest pain, and dyspnea, which are variable in severity. The evaluation of CAP severity is crucial for the selection of appropriate location of treatment. Among multiple severity assessment tools for CAP, CURB65 (consciousness, urea nitrogen, respiratory rate, blood pressure, and age) stands out for its simplicity and efficacy and has been recommended by major CAP guidelines worldwide (17–20). Moreover, the five components of CURB65 are mostly objective parameters, which make it unlikely to suffer from the subjective interpretation of both patients and clinicians. These features make CURB65 a potential candidate to be applied in the CIP grade. So far, the potential utility of CURB65 in CIP has not been reported yet. Therefore, the aim of the current study is to explore preliminarily if CURB65 could predict the mortality in NSCLC patients with CIP.

## Methods

### Ethical approval

The present study was a retrospective study conducted in a Chinese hospital (Second Affiliated Hospital of Zhejiang University School of Medicine, China). Ethical approval was sought and granted by the Ethics Committee of the Second Affiliated Hospital of Zhejiang University School of Medicine (Approval Number: 2022-0240). As the non-interventional retrospective study was determined to be no greater than minimal risk, the Ethics Committee of the Second Affiliated Hospital of Zhejiang University School of Medicine issued a waiver of informed consent. Patient data privacy and confidentiality were maintained as this study was conducted in compliance with the ethical standards of the Declaration of Helsinki.

### Study population

The NSCLC patients with CIP were identified from the consultation records of the lung cancer multidisciplinary team in the study hospital from 1 January 2019 to 31 December 2021. The multidisciplinary team consisted of multiple subspecialties, including oncology, pulmonology, radiotherapy, radiology, thoracic surgery, and infectious disease, among others. Before consulting the multidisciplinary team, all patients underwent chest computer tomography (CT) imaging. For identifying CIP patients, the multidisciplinary team considered patients who developed dyspnea or other respiratory symptoms (including fever, cough, sputum production, etc.) after use of ICIs, along with the presence of new radiographic infiltration on CT and lack of evidence of lung infection or other alternative etiologies (tumor progression, radiation pneumonitis, diffuse alveolar hemorrhage, heart failure, etc.). Therapies included programmed cell death protein 1 (PD-1) ICIs and programmed cell death receptor ligand-1 (PD-L1) ICIs with or without additional agents, and tumor types included NSCLC only. The list of lung cancer patients receiving at least one dose of ICIs during the study period was acquired from the Electronic Medical Record System.

CIP was graded by the lung cancer multidisciplinary team according to the irAE guideline published in 2018 by the American Society of Clinical Oncology (ASCO) (13), as follows: G1: asymptomatic, confined to one lobe of the lung or 25% of lung parenchyma, clinical or diagnostic observations only; G2: symptomatic, involves more than one lobe of the lung or 25%–50% of lung parenchyma, medical intervention indicated, limiting instrumental activities of daily living (ADL); G3: severe symptoms, hospitalization required, involves all lung lobes or 50% of lung parenchyma, limiting self-care ADL, oxygen indicated; G4: life-threatening respiratory compromise, urgent intervention indicated (intubation).

### CURB65

The CURB65 score was calculated as described before, by a pulmonologist (LXX) who was blinded to patients’ ICI treatment history (18). One point was designated for each of confusion, blood urea >7 mmol/l, respiratory rate >30/min, low systolic (<90 mm Hg) or diastolic (≤60 mm Hg) blood pressure, and age ≥65 years. The score ranged from 0 to 5 in this scoring system, with a higher score indicating increasing disease severity.

### Data collection

Detailed clinical data were collected retrospectively, including demographic characteristics, tumor history and prior treatment history, types of ICIs, clinical manifestations of CIP, lab test results, results of chest imaging and bronchoscopy, and the treatment outcomes of CIP. For patients who received corticosteroids for CIP treatment, a cumulative hydrocortisone-equivalent dose was calculated. The chest CT scan radiographic patterns were classified by an experienced radiologist (LHW) as described previously (21), including organizing pneumonia (OP)-like pattern, non-specific interstitial pneumonia (NSIP)-like pattern, diffuse alveolar damage (DAD)-like pattern, hypersensitivity pneumonitis (HP)-like pattern, and bronchiolitis-like pattern. Survival status was assessed by medical records and phone call during early April 2022.

### Data analysis

The results were analyzed using IBM SPSS Statistics 20. Continuous data were presented as the mean with standard deviation (SD) or median with interquartile range (IQR), depending on the distribution of data. Variables were compared using the unpaired Student’s t-test, Welch t-test, or Wilcoxon rank-sum test with continuity correction, depending on data normality and homogeneity of variance. Categorical data were presented as absolute value and percentage and analyzed using chi-square test or Fisher’s exact test according to test assumptions. Pearson’s correlation analysis was used for analyzing the correlation between variables. Statistical significance was set at p < 0.05.

## Results

**Figure 1:**
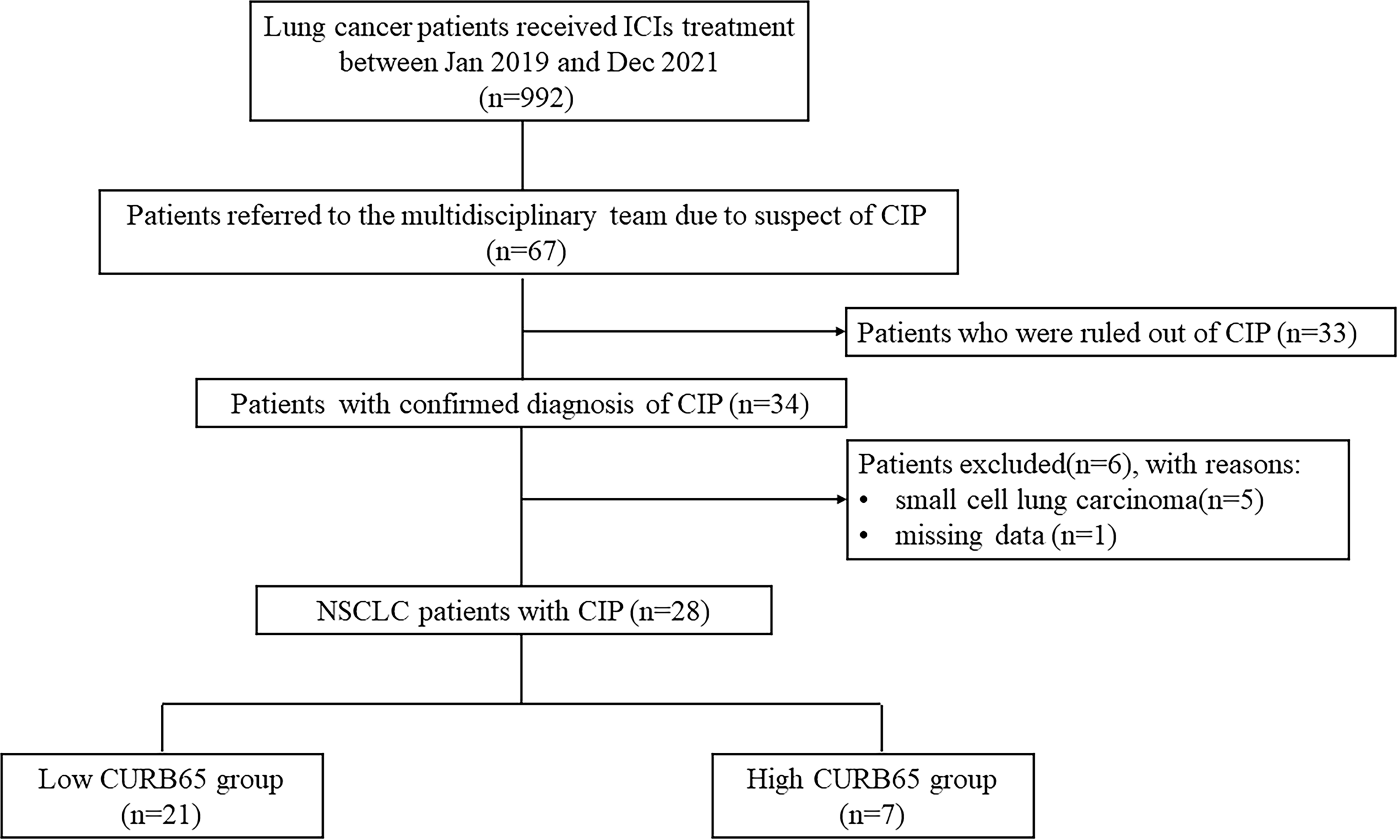
Flowchart of the study population. ICIs, immune checkpoint inhibitors; CIP, checkpoint inhibitor-associated pneumonitis; NSCLC, non-small cell lung carcinoma; CURB65, consciousness, urea nitrogen, respiratory rate, blood pressure, and age.

Between 1 January 2019 and 31 December 2021, a total number of 992 lung cancer patients received at least one dose of ICIs in the study hospital. A number of 67 patients suspected of CIP were referred to the multidisciplinary team by their attending doctors. The multidisciplinary team confirmed the diagnosis of CIP in 34 patients and ruled out CIP in 33 patients. Of all 34 patients with CIP, five patients were excluded due to subtype of small cell lung carcinoma, and one patient was excluded due to missing data. Therefore, a final number of 28 NSCLC patients with CIP were included in the current study (**Figure 1**). Patients were classified into two groups for further analysis according to CURB65: low-CURB65 group (for patients with CURB65 score of 0–1, n = 21) and high-CURB65 group (for patients with CURB65 score ≥2, n = 7).

### Baseline features

The baseline demographics, comorbidities, and lung function test results between two groups were compared (**Table 1**). The high-CURB65 group had significantly higher age than the low-CURB65 group (71.29 ± 3.59 vs. 66.14 ± 5.76, p = 0.037). The age difference could be explained by the fact that CURB65 had a component of age ≥65. All the patients in the high-CURB65 group had either ever smoking history (85.7%) or current smoking history (14.3%), which were different to the patients in the low-CURB65 group, although without statistical significance (p = 0.051). The gender, body mass index (BMI), comorbidities, and lung function test results were similar between two groups.

**Table 1:** Baseline demographics, comorbidities, and lung function test results.

Variables	Low-CURB65 group (n = 21)	High-CURB65 group (n = 7)	p
**Age**	66.14 (5.79)	71.29 (3.59)	0.037
**Male**	17 (81%)	7 (100%)	0.212
**BMI**	22.71 (3.57)	21.49 (1.84)	0.400
**Smoking history**			0.051
Ever	7 (33.3%)	6 (85.7%)	
Current	9 (42.9%)	1 (14.3%)	
Never	5 (23.8%)	0	
**Pack-years**	45 (30.00, 60.00)	50 (30.00, 60.00)	0.824
**Comorbidities**			
COPD	5 (23.8%)	3 (42.9%)	0.334
Asthma	0	0	
ILD	3 (14.3%)	1 (14.3%)	1.000
Hypertension	7 (33.3%)	3 (42.9%)	0.649
Diabetes mellitus	1 (4.8%)	1 (14.3%)	0.397
**Lung function test^#^**			
FEV1	1.80 (0.70)	1.75 (0.56)	**—**
FEV1% predicted	74.03 (18.86)	68.75 (15.71)	**—**
FVC	2.52 (0.98)	2.52 (0.66)	**—**
FVC % predicted	81.30 (20.88)	75.80 (11.84)	**—**
DLCO % predicted	4.04 (1.14)	4.36 (0.99)	**—**
No spirometry performed	13 (61.9%)	3 (42.9%)	**—**

All data are presented as no. (%), median (interquartile range), or mean (standard deviation).

BMI, body mass index; COPD, chronic obstructive pulmonary disease; ILD, interstitial lung disease; FEV1, forced expiratory volume in one second; FVC, forced vital capacity; DLCO, carbon monoxide diffusing capacity.

^#^ The statistical analysis was not performed due to a very small sample size.

### Lung cancer history and ICI treatment

Lung cancer history and ICI treatment were also analyzed (**Table 2**). The low-CURB65 group had one-third of patients with adenocarcinoma and two-thirds with squamous cell carcinoma, and the high-CURB65 group had three patients (42.9%) with adenocarcinoma, three patients (42.9%) with squamous cell carcinoma, and one patient (14.3%) with large cell carcinoma. The performance status and stage of patients were similar between two groups. ICIs were used predominantly in the second-line setting for both groups, because most patients had received chemotherapy, thoracic radiotherapy, or thoracic surgery before ICI initiation. The commonly used ICIs in the low-CURB65 group were pembrolizumab(19%), camrelizumab (38.1%), and tislelizumab (14.3%). In the high-CURB65 group, the commonly used ICI agents were camrelizumab (57.1%) and tislelizumab (28.6%). The median number of ICI cycles received was 5.0 (2.0–15.0) in the low-CURB65 group and 4.0 (2.0–7.0) in the high-CURB65 group. PD-L1 expression was determined from histologic specimens in nine patients (42.8%) of the low-CURB65 group and two patients (28.5%) of the high-CURB65 group, respectively. ICIs were commonly used in combination with chemotherapy in both groups (90.5% and 85.7%, respectively).

**Table 2:** Lung cancer history and ICI treatment.

Variables	Low-CURB65 group (n = 21)	High-CURB65 group (n = 7)	p
**Histology**			0.163
Adenocarcinoma	7 (33.3%)	3 (42.9%)	
Squamous cell carcinoma	14 (66.7%)	3 (42.9%)	
Large cell carcinoma	0	1 (14.3%)	
**Performance status**			0.599
0	3 (14.3%)	2(28.6%)	
1	16 (76.2%)	5 (71.4%)	
2	2 (9.5%)	0	
**Stage**			1.000
III	10 (47.6%)	3 (42.9%)	
IV	11 (52.4%)	4 (57.1%)	
**Prior cancer treatment**			
Thoracic surgery	10 (47.6%)	4 (57.1%)	0.663
Thoracic radiotherapy	5 (23.8%)	0	0.076
Chemotherapy	7 (33.3%)	2 (28.6%)	0.815
**ICIs**			
Pembrolizumab	4 (19%)	0	**—**
Camrelizumab	8 (38.1%)	4 (57.1%)	**—**
Tislelizumab	6 (28.6%)	2 (28.6%)	**—**
Others	3 (14.3%)	1 (14.3%)	**—**
**ICI cycles**	5.0 (2.0, 15.0)	4.0 (2.0, 7.0)	0.455
PD-L1 expression status^a^			
Positive^b^	5 (23.8%)	0	**—**
Negative	4 (19.0%)	2 (28.5%)	**—**
Not assessed	12 (57.2%)	5 (71.5%)	**—**
**Concurrent treatment with ICIs**			
Chemotherapy	19 (90.5%)	6 (85.7%)	0.724
None	2 (9.5%)	1 (14.3%)	0.204

All data are presented as no. (%), median (interquartile range), or mean (standard deviation).

ICIs, immune checkpoint inhibitors; PD-L1, programmed cell death-ligand 1.

a The statistical analysis was not performed due to the small sample size.

b If PD-L1 expression was >1%.

### Kaplan–Meier analysis of mortality

**Figure 2:**
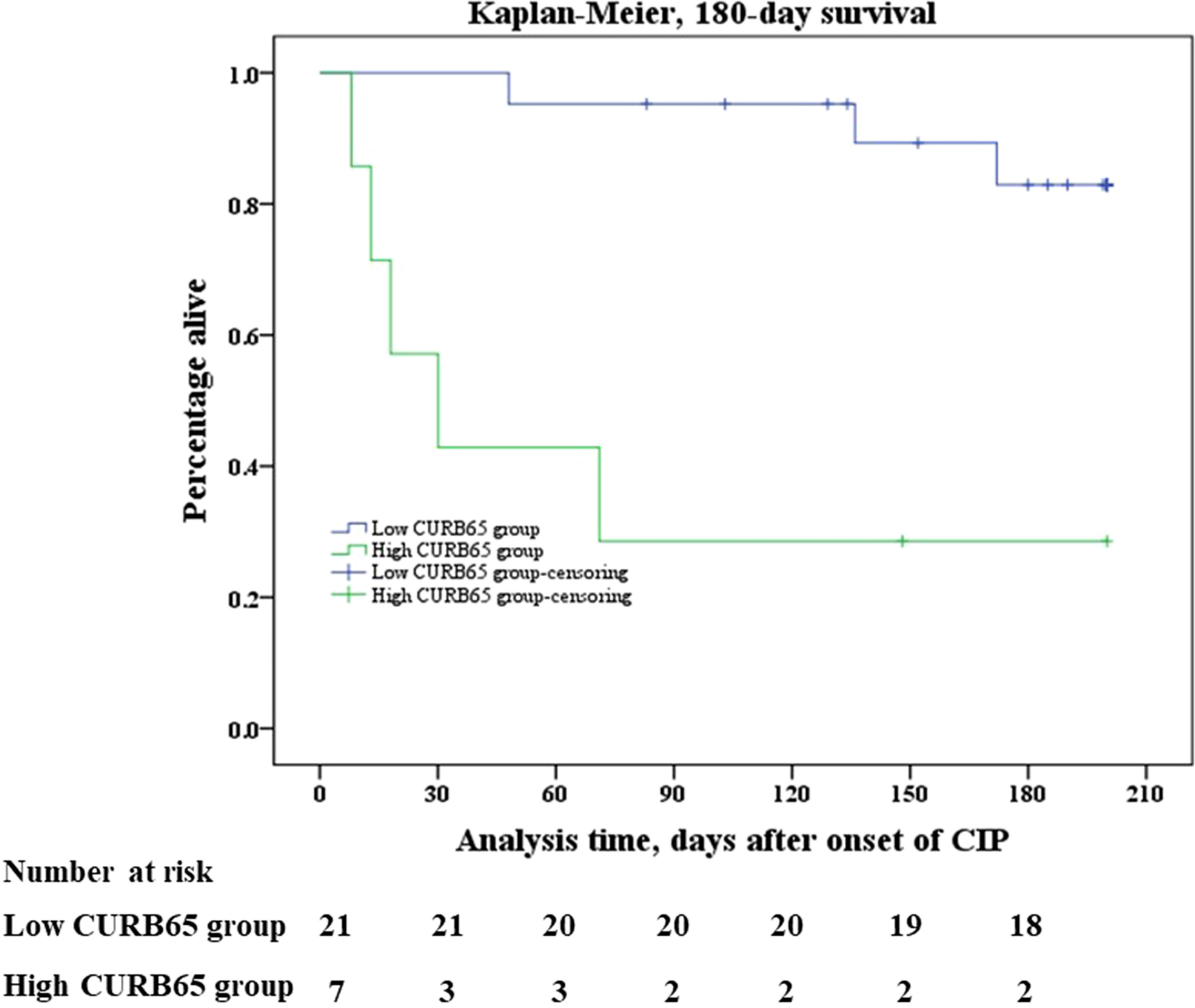
Kaplan–Meier survival analysis. Kaplan–Meier analysis of survival in 180 days after onset of CIP showed that mortality was significantly higher in the high-CURB65 group than in the low-CURB65 group (log rank, p < 0.001). CURB65, consciousness, urea nitrogen, respiratory rate, blood pressure and age; CIP, checkpoint inhibitor-associated pneumonitis.

Kaplan–Meier analysis identified a significant difference between two groups in all-cause mortality after onset of CIP. Mortality was significantly higher in the high-CURB65 group than in the low-CURB65 group, up to 180 days after onset of CIP (log rank, p < 0.001) (**Figure 2**). The mortality was consistently higher in the high-CURB65 group (30-day: 57.1% vs. 0; 90-day: 71.4% vs. 4.76%; 180-day:71.4% vs. 14.29%).

The median follow-up time of the study population was 178.5 days (88.0–261.8 days). There were three death events in the low-CURB65 group and five death events in the high-CURB65 group during the follow-up. Six patients died in the hospital, and their death records showed that their cause of death was CIP. One patient died 1 day later after hospital discharge, and the medical records showed that the patient was in critical state due to CIP before discharge. The relatives required the discharge because according to their local custom, people should die at home. The cause of death of the last patient could not be verified. There were 18 censored cases in the low-CURB65 group and the censored cases in the high-CURB65 group, respectively.

### CIP characteristics and treatment

The median time to CIP diagnosis from initial ICI treatment was 145.0 days (44.5–333.5 days) for the low-CURB65 group and 139.0 days (45.0–168.0 days) for the high-CURB65 group (**Table 3**). Two patients (9.5%) in the low-CURB65 group had severe CIP (ASCO grade ≥3), and more than half of patients in the high-CURB65 group had severe CIP (p = 0.0008). The symptoms were similar between two groups, although the high-CURB65 group tended to have more patients with fever without statistical significance. The high-CURB65 group had higher C-reactive protein than the low-CURB65 group, but without statistical significance (94.5 ± 83.7 vs. 52.2 ± 45.8, p = 0.11). The high-CURB65 group also had significantly higher D-dimer (p = 0.002).

**Table 3:** CIP characteristics and treatment.

Variables	Low-CURB65 group (n = 21)	High-CURB65 group (n = 7)	p
**Onset time of CIP**	145.0 (44.5, 333.5)	139.0 (45.0, 168.0)	0.490
**ASCO grade**			0.008
G1–2	19 (90.5%)	3 (42.9%)	
G3–4	2 (9.5%)	4 (57.1%)	
**Symptoms**			
Fever	4 (19.0%)	4 (57.1%)	0.053
Cough	9 (42.9%)	3 (42.9%)	1.000
Sputum production	8 (36.1%)	3 (42.9%)	0.823
Chest pain	1 (4.8%)	0	0.557
Dyspnea	11 (52.4)	7 (100%)	0.663
**Blood test results**			
CRP	52.2 (45.8)	94.5 (83.7)	0.110
D-dimer	930.0 (480.0, 1860.0)	3560.0 (2410.0, 8950.0)	0.002
Albumin	34.1 (5.2)	29.7 (7.0)	0.170
White blood cell count	7.02 (2.23)	9.50 (5.93)	0.119
Neutrophil count	5.40 (2.01)	7.67 (5.31)	0.112
Lymphocyte count	0.95 (0.39)	1.02 (0.46)	0.541
Eosinophil count	0.17 (0.27)	0.12 (0.09)	0.613
Hemoglobin	116.20 (18.00)	105.86 (12.47)	0.174
Platelet count	223.40 (69.33)	168.71 (100.63)	0.123
**Corticosteroid treatment**			
Use of corticosteroids	16 (76.2%)	7 (100%)	0.154
Cumulative dose of corticosteroids	1240.00 (850.00, 2400.00)	3600.00 (750.00, 7000.00)	0.111
Daily dose of corticosteroids	200.00 (200.00, 291.67)	257.14 (148.97, 400.00)	0.042
Duration of corticosteroid use	6.00 (5.00, 10.50)	8.00 (3.00, 23.00)	0.614
**Other treatment**			
Antibiotics	14 (66.7%)	5 (71.4%)	0.815
IVIG	0	2 (28.6%)	0.056
Non-invasive ventilation	0	1 (14.3%)	0.250
Invasive ventilation	0	1 (14.3%)	0.250

All data are presented as no. (%), median (interquartile range), or mean (standard deviation).

CIP, checkpoint inhibitor-associated pneumonitis; ASCO, American Society of Clinical Oncology; CRP, C-reactive protein; IVIG, intravenous immunoglobins.

The patients in the high-CURB65 group received more aggressive treatment. Corticosteroids were used in 76.2% of patients in the low-CURB65 group and 100% of patients in the high-CURB65 group. The high-CURB65 group tended to have a higher cumulative hydrocortisone-equivalent dose of corticosteroids, daily dose of corticosteroids, and duration of corticosteroid use, but statistical significance was only detected for the daily dose of corticosteroids (p = 0.042). The high-CURB65 group was more inclined to receive additional immunosuppressants and respiratory support. In the high-CURB65 group, besides corticosteroids, one patient received both intravenous immunoglobin (IVIG) and non-invasive ventilation, and another patient received both IVIG and invasive ventilation.

### Radiographic appearances of CIP

During the evaluation for CIP, all patients underwent chest CT imaging. The low-CURB65 group had 11 patients (52.4%) who presented with bilateral involvement, and the high-CURB65 group had six patients (85.7%) (**Table 4**). The low-CURB65 group had 3.0 (2.0–4.5) lobes involved, and the high-CURB65 group had 4.0 (4.0–5.0) lobes. The high-CURB65 group had a significantly higher proportion of patients with pleural effusion than the low-CURB65 group (71.8% vs. 14.3%, p = 0.004). The overall radiographic pattern profile of CIP was similar two groups, which both showed a predominant OP-like pattern.

**Table 4:** Radiographic appearances of CIP.

Variables	Low-CURB65 group (n = 21)	High-CURB65 group (n = 7)	p
**Bilateral involvement**	11 (52.4%)	6 (85.7%)	0.118
**Number of lobes involved**	3.0 (2.0, 4.5)	4.0 (4.0, 5.0)	0.242
**Pleural effusion**	3 (14.3%)	5 (71.8%)	0.004
Overall pattern of CIP^a^			0.250
OP-like pattern	15 (71.4%)	3 (42.9%)	
NSIP-like pattern	2 (9.5%)	1 (14.3%)	
DAD-like pattern	2 (9.5%)	3 (42.9%)	
HP-like pattern	2 (9.5%)	0	
Bronchiolitis-like pattern	1 (4.8%)	0	

All data are presented as no. (%).

CIP, checkpoint inhibitor-associated pneumonitis; OP, organizing pneumonia; NSIP, nonspecific interstitial pneumonia; DAD, diffuse alveolar damage; HP, hypersensitivity pneumonitis.

a One patient in the low-CURB65 group presented both OP-like and NSIP-like patterns.

### Correlation between CURB65 score and ASCO grade of CIP

**Figure 3:**
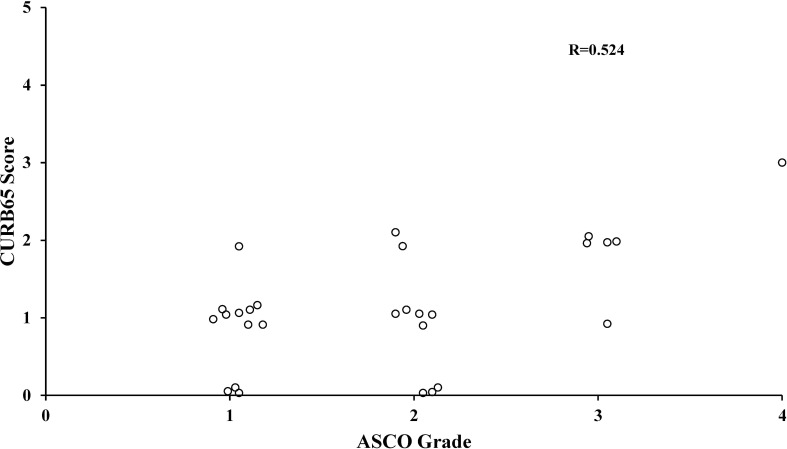
Correlation between CURB65 score and ASCO grade of CIP. The Pearson correlation analysis showed a moderately positive linear correlation between CURB65 and ASCO grade of CIP. CURB65, consciousness, urea nitrogen, respiratory rate, blood pressure and age; CIP, checkpoint inhibitor-associated pneumonitis. ASCO, American Society of Clinical Oncology.

The scatter plots showed a moderate positive linear correlation between CURB65 and ASCO grade of CIP (**Figure 3**). The Pearson correlation coefficient R between the two variables was 0.524 (p = 0.004).

## Discussion

There was a lack of objective and evidence-based tool to assess the severity of CIP. To our knowledge, the current study was the first study to explore preliminarily if CURB65 could predict the mortality in NSCLC patients with CIP. Our study showed that CURB65 accurately stratified the risk of mortality in 180 days after onset of CIP. The high-CURB65 group had significantly more severe CIP and received more aggressive treatment. CURB65 was moderately correlated with the ASCO grade of CIP. CURB65 had the potential to be a useful clinical predictive tool, when used in conjunction with ASCO grade, to risk-stratify patients and assist in clinical decision making and personalized medicine approaches in NSCLC patients with CIP. However, further prospective studies were warranted to verify its efficacy.

CURB65 was first derived and validated by Lim *et al.* in 2003 (18). It was based on the modified British Thoracic Society severity assessment tool which used clinical features to identify severe CAP patients at high risk of mortality. They found that CURB65, based on information available at initial hospital assessment, enabled CAP patients to be stratified according to increasing risk of mortality (score 0, 0.7%; score 1, 2.1%; score 2, 9.2%; scores 3–5, 15%–40%). Besides mortality, piling evidence validated the effectiveness of the CURB-65 score in predicting various CAP outcomes including disease complications, hospitalization or intensive care unit (ICU) admission, duration of hospital or ICU stay, intensive respiratory or vasopressor support, mechanical ventilation, and treatment failure (22). The evidence base for the CURB65 score in CAP was robust and continued to increase. Moreover, CURB65 used only five items which required no special tests and was practical for calculations. This simplicity made it easier to be popularized and applied in practice. So CURB65 had been universally recommended by major CAP guidelines to assist the clinical judgment for determination of the site of care (17, 19, 20). To the best of our knowledge, the potential utility of CURB65 in CIP has not been reported yet.

Our study showed for the first time that CURB65 accurately stratified the risk of mortality in NSCLC patients with CIP. Compared with the low-CURB65 group, we found that there was a consistently increased risk of death in the high-CURB65 group. As far as we knew, there was no similar report before. This finding indicated that CURB65 might be used to identify patients at high risk of death, and more aggressive interventions might be warranted for those patients. This finding should be interpreted with caution, because of the small sample size. However, all published studies about CIP in lung cancer had a relatively small sample size, and most studies were case reports or case series. A study by Atchley *et al.* included 30 lung cancer patients with CIP, and another study by Huang *et al.* recruited 32 NSCLC patients with CIP (23, 24). Our sample size was comparable to the previous studies. Besides the small sample size, the low-CURB65 group had more patients than the high-CURB65 group (21 vs. 7). The unbalanced sample size of the two groups may also lead to bias of the analysis results. It was possible that the unbalance groups may cause the overestimation of mortality difference between low-CURB65 and high-CURB65 groups. However, the mortality difference (about fivefold) was so significant that the principal findings of the current study was unlikely to be caused by biased information. Future prospective multicenter studies with a large sample size and more balanced groups were needed to further verify the efficacy of CURB65.

It remained unknown whether CURB65 was a predictor specific to CIP or just a general prognostic factor for lung cancer. On the one hand, our findings tended to support that CURB65 was a predictor specific to CIP. Most clinical studies of CAP used 30-day mortality as a clinical end point, because deaths that occurred within 30 days were most likely attributed to CAP (22). Therefore, it could be plausibly argued that in patients with CIP, deaths that occurred within 30 days after onset of CIP were most likely attributed to CIP. Our study showed that the high-CURB65 group had a significantly higher 30-day mortality than the low-CURB65 group (57.1% vs. 0). Therefore, the high mortality of the high-CURB65 group within 30 days was most likely to be caused by CIP instead of lung cancer. On the other hand, CURB65 used all objective parameters, which were not specific to CIP. It was reported that CURB65 was associated with advanced age, hypertension, overweight/obesity, kidney failure, hypoxemia, requirement for mechanical ventilation, or onset of respiratory distress in patients hospitalized with Coronavirus Disease 2019 (COVID-19) (25).Thus, it was very likely to have some non-CIP patients with high CURB65. In order to answer the abovementioned question, future studies with the aim to explore the predictive value of CURB65 in lung cancer patients without CIP were warranted.

The current study revealed that the high-CURB65 group had significantly more severe CIP (57.1% vs. 9.5%). It was reported that the prognosis of severe CIP was worse than non-severe CIP. A study by Tone *et al.* revealed that patients with severe CIP had significantly shorter progression-free survival (PFS) and overall survival (OS) than patients with non-severe CIP (26). Univariate analysis further confirmed that complication with severe grade CIP was significantly associated with poor PFS and OS. A review by Zhang *et al.* included and analyzed 44 occurrences of CIP in patients with NSCLC, which were all published in case reports and case series (11). Although not powered to detect statistical significance, it was reported that severe CIP had significantly higher mortality than non-severe CIP (57.14%–64.29% vs. 14.29%). Therefore, the high proportion of severe CIP may at least partially explain the high mortality in the high-CURB65 group.

Moreover, the current study found that there was a trend to a higher proportion of ever or current smokers in the high-CURB65 group than the low-CURB65 group, with borderline significance. So far, there were limited reports about the role of smoking history in CIP, which were all from retrospective studies. First, history of smoking may increase the risk of CIP (11). Second, smoking history was a risk factor for severe CIP. In a study conducted by Chen *et al.*, patients with severe pneumonitis had a higher likelihood of being current or former smokers than patients with non-severe pneumonitis (100% vs. 77%, p = 0.007) (27). Therefore, our findings were in agreement with previous reports. Future prospective studies were warranted to further explore the role of smoking history in CIP.

Our study found that the patients with high CURB65 received more aggressive treatment. Current guidelines for irAE recommended that management of CIP should be based on CIP grade (13–15). Corticosteroids were recommended as the primary therapy approach, although in mild cases, holding ICIs might suffice. The suggested dose of corticosteroids tended to increase with the grade of CIP, and additional immunosuppressants and empirical antibiotics were recommended for severe CIP. In the current study, corticosteroids were more likely to be used in the high-CURB65 group than in the low-CURB65 group (100% vs. 76.2%). Furthermore, there was a tendency toward a higher dose of corticosteroids and more use of IVIG and respiratory support in the high-CURB65 group. This demonstrated that in the current study, the management was dependent on the severity of CIP according to CURB65. This fact was in agreement with the recommendations by the irAE guidelines that management of CIP should be based on grade.

The current study also revealed that CURB65 was moderately correlated with the ASCO grade of CIP, with a Pearson correlation coefficient R of 0.524. CURB65 evaluates the severity with five objective parameters, and the ASCO grade evaluates the severity by a combination of subjective clinical symptoms and imaging manifestations (13). Therefore, by assessing the severity of CIP from different perspectives, the two scoring systems did not fully agree with each other, which was not unexpected. The moderate correlation indicated that they might complement each other. Of notice, in the current study, there were three patients with a low ASCO grade in the high-CURB65 group. The severity of CIP in these patients might be underestimated, which led to insufficient and inappropriate treatment. This might at least partially contribute to the result that two of the three patients died within 30 days. For these patients, more aggressive interventions might improve the prognosis. Therefore, CURB65 might complement the ASCO grade in the assessment and prediction of mortality. Especially for the patients with a low ASCO grade but high CURB65 score, more aggressive interventions might be warranted.

This study had several limitations. First, the present study was a retrospective study, which came with many inherent limitations. The current retrospective study could not establish a cause–effect relationship between CURB65 and mortality. The retrospective nature of this study was also prone to biases from missing data and reliance on documentation available for review. Second, patients with mild CIP may be under-represented in the current study. Because those patients had no symptom or mild symptom, the clinicians were less likely to refer these patients to the lung cancer multidisciplinary team for consultation. Third, the paradigm of ICI use had shifted since the initial use of these agents, so our study population could not represent the present profile of patients with ICI treatment.

## Conclusion

The current study provided preliminary evidence to support the use of the CURB65 score in predicting mortality in NSCLC patients with CIP for the first time. CURB65 accurately stratified the risk of mortality in 180 days after onset of CIP. The high-CURB65 group had significantly more severe CIP and received more aggressive treatment. CURB65 was moderately correlated with the ASCO grade of CIP. CURB65 might complement the ASCO grade in the assessment and prediction of mortality in NSCLC patients with CIP.

## Data availability statement

The raw data supporting the conclusions of this article will be made available by the authors, without undue reservation.

## Ethics statement

This study was reviewed and approved by Ethics Committee of Second Affiliated Hospital of Zhejiang University School of Medicine. The ethics committee waived the requirement of written informed consent for participation.

## Author contributions

YM and WL contributed to the conception and design of the study. FL contributed to the writing of the manuscript, design of the study, and statistical analysis. BF and LW contributed to the writing of the manuscript, retrieval of data, and organization of the database. LX and TZ contributed to the retrieval of data. All authors contributed to the manuscript revision and read and approved the submitted version.

## Funding

This study is supported by the National Natural Science Foundation of China [No. 81500061].

## Conflict of interest

The authors declare that the research was conducted in the absence of any commercial or financial relationships that could be construed as a potential conflict of interest.

## Publisher’s note

All claims expressed in this article are solely those of the authors and do not necessarily represent those of their affiliated organizations, or those of the publisher, the editors and the reviewers. Any product that may be evaluated in this article, or claim that may be made by its manufacturer, is not guaranteed or endorsed by the publisher.

## Abbreviations

ICIs, immune checkpoint inhibitors; NSCLC, non-small cell lung carcinoma; irAEs, immune-related adverse events; CIP, checkpoint inhibitor-associated pneumonitis; CAP, community-acquired pneumonia; CURB65, consciousness, urea nitrogen, respiratory rate, blood pressure and age; PD-1, programmed cell death protein 1; PD-L1, programmed cell death receptor ligand-1; ASCO, American Society of Clinical Oncology; ADL, activities of daily living; OP, organizing pneumonia; NSIP, non-specific interstitial pneumonia; DAD, diffuse alveolar damage; HP, hypersensitivity pneumonitis; SD, standard deviation; IQR, median with interquartile range; BMI, body mass index; IVIG, intravenous immunoglobin; ICU, intensive care unit; COVID-19, Coronavirus Disease 2019; PFS, progression-free survival; OS, overall survival.
